# Wheat Can Access Phosphorus From Algal Biomass as Quickly and Continuously as From Mineral Fertilizer

**DOI:** 10.3389/fpls.2021.631314

**Published:** 2021-01-28

**Authors:** Lisa Mau, Josefine Kant, Robert Walker, Christina M. Kuchendorf, Silvia D. Schrey, Ute Roessner, Michelle Watt

**Affiliations:** ^1^Institute of Bio- and Geoscience - IBG-2: Plant Science, Forschungszentrum Jülich GmbH, Jülich, Germany; ^2^Faculty of Agriculture, University of Bonn, Bonn, Germany; ^3^School of BioSciences, The University of Melbourne, Melbourne, VIC, Australia

**Keywords:** algae fertilizer, mass balance, phosphorus, plant growth, resource efficiency, wheat

## Abstract

Algae can efficiently take up excess nutrients from waterways, making them a valuable resource potentially capable of replacing synthesized and mined fertilizers for agriculture. The capacity of algae to fertilize crops has been quantified, but it is not known how the algae-derived nutrients become available to plants. We aimed to address this question: what are the temporal dynamics of plant growth responses to algal biomass? to better propose mechanisms by which plants acquire nutrients from algal biomass and thereby study and promote those processes in future agricultural applications. Data from various sources were transformed and used to reconstruct the nutrient release from the algae *Chlorella vulgaris* and subsequent uptake by wheat (*Triticum aestivum* L.) (as reported in Schreiber et al., 2018). Plants had received 0.1x or 1x dried algae or wet algae, or zero, 0.1x or 1x mineral fertilizer calculated from agricultural practices for P application and grown to 55 days in three soils. Contents of P and other nutrients acquired from algae were as high as from mineral fertilizer, but varied based on moisture content and amount of algae applied to soils (by 55 days after sowing plants with 1x mineral fertilizer and 1x dried algae had 5.6 mg P g DW_shoot_; 2.2-fold more than those with 0 or 0.1x mineral fertilizer, 0.1x dried algae and wet algae, and 1x wet algae). Absolute and relative leaf area growth and estimated P uptake rates showed similar dynamics, indicating that wheat acquires P from algae quickly. A model proposes that algal fertilizer promotes wheat growth after rapid transformation in soil to inorganic nutrients. We conclude theoretically that phosphorus from algal biomass is available to wheat seedlings upon its application and is released gradually over time with minor differences related to moisture content on application. The growth and P uptake kinetics hint at nutrient forms, including N, and biomass stimulation worthy of research to further exploit algae in sustainable agriculture practices. Temporal resolved phenotype analyses in combination with a mass-balance approach is helpful for understanding resource uptake from recycled and biofertilizer sources by plants.

## Introduction

The objective of this paper was to resolve the kinetics of how quickly nutrients including phosphate from algae biomass becomes available and is taken up by wheat plants compared to mineral nutrient sources. Identified patterns will serve as scheme aiming at using algae biomass as fertilizers in future agricultural applications.

### Background for Algal Biomass and Its Application to Crops

Freshwater algae are ubiquitous and can be grown in media of varying quality, from sterile through to different types of wastewater. They require only water, light, nutrients, and CO_2_. Environmental limitations such as growing temperature, pH, and culture densities depend on species. Algae are highly effective producers of organic material and do not compete with agricultural production for arable land. Multiple purposes for algae have been identified including producers of fuel, oil, gas or direct combustion, food and feed, pharmaceutical compounds, plastic, polymer compounds, and fertilizer. While most of these applications are feasible, the majority are not currently economically viable.

The production of algae biomass as a fertilizer has dual value; the use of algae for the purification of wastewater coupled with the application of obtained biomass as fertilizer. The underlying concept is circular economy fertilization and has been reviewed before (Solovchenko et al., 2016). Various methods have been used to recover nutrients from wastewater for agricultural purposes, including sewage sludge (directly or treated) (Duboc et al., 2017), precipitation of nutrients as struvite (Rahman et al., 2011), and algae cultivation. Besides approaches to mainly clean water with algae cultivation (e.g., Powell et al., 2008; Shilton et al., 2012) others were aimed to specifically produce algae biomass from simulated waste water (Gimondo et al., 2019) or real waste water (Mulbry et al., 2005) or different purchased species (Alobwede et al., 2019) to be used as a fertilizer on different plant species. The recycling of nutrients with algae can help to reduce organic contamination from sources such as bacteria and viruses, pollutants such as antibiotics, and nutrients that cause problems with wastewater (reviewed in Bloem et al., 2017). Other benefits, such as the specific accumulation of heavy metals shown in some species, remain to be investigated (Safi et al., 2014).

The ability of algae to accumulate nutrients, directly and specifically, make them a valuable vector for recycling nutrients in agricultural systems. Algae can store excess phosphorus (P) and nitrogen (N) inside their cells resulting in high amounts of P and N that can be stored in vacuoles or the cytosol (Ismagulova et al., 2018). Excess supply of phosphate, or similar P sources, can be stored as polyphosphate granules in storage bodies (Miyachi et al., 1964). Additional nitrogen is stored in form of crystalline guanine rich inclusions (Moudříková et al., 2017).

Phosphorus and N fertilization increase the risks for environmental pollution. However, they are the most essential macroelements for crop growth and therefore their acquisition and application are critical political and resource issues for global food and environment security (Conley et al., 2009). Although P in general is quite abundant in our earth crust (Shen et al., 2011), it limits agricultural yields because it is mainly not in the phosphate form; the form generally considered the only form taken up by plants (MacDonald et al., 2011). Phosphate is largely immobile in soils and is easily adsorbed or precipitated, or organically incorporated into other organisms (Shen et al., 2011). Plants need P in form of phosphate to produce membranes, RNA and DNA, as well as proteins, it is involved in energy metabolism (e.g., for ATP), and the regulation of enzyme activities. The chemical properties of phosphate make it an irreplaceable and important nutrient for plants, but also a scarce resource that introduces competition and investment of resources in the rhizosphere (Steidinger et al., 2015). N and P have been shown to influence each other (reviewed in Güsewell, 2004). N is, compared to phosphate, more mobile in soils and can become gaseous and evaporate. Crop plants such as wheat acquire N in the form of nitrate and ammonium organic forms, such as e.g., amino acids, can also be used by plants native to natural ecosystems (Turnbull et al., 1996). Nitrate uptake is more energy consumptive and ammonium can therefore be favored, but it is species (and pH) dependent (Falkengren-Grerup, 1995). The uptake of organic forms of P and N directly by plant roots has been rarely measured (Turnbull et al., 1996; Richardson et al., 2000; Paungfoo-Lonhienne et al., 2008).

Several studies have analyzed plant responses to algal biomass (Mulbry et al., 2005; Grzesik and Romanowska-Duda, 2014; Mukherjee et al., 2015, 2016; Kholssi et al., 2018; Schreiber et al., 2018). Application of cyanobacteria has a positive effect on maize under mild drought and slightly elevated temperature conditions (Piotrowski et al., 2016). Foliar application of both, cyanobacteria and green algae *Chlorella*, were shown to improve growth and metabolic activity in willow (Grzesik et al., 2017). Mulbry et al., 2005 found that cucumber and maize seedlings acquired N from algae that had been grown in diluted manure. Mukherjee et al., 2016 showed that rice seedlings had greater height, leaf width and biomass when supplied with different algae species. Polyphosphate was abundant in these algae. Plants receiving algae in unsterile soils, increased phosphate over time although at a rate twice as slow as that in plants provided with mineral phosphate. Evidence of slower P release from algae than mineral fertilizer in rice agrees with previous work of this group (Mukherjee et al., 2015). Grzesik and Romanowska-Duda, 2014 treated maize seeds with *Chlorella sp.*, found positive healthy effects for 14 days in seedlings, and proposed a signaling mechanism rather than a nutrient mechanism. Kholssi et al., 2018 grew wheat for 2 weeks in solution with *Chlorella sorokiniana* and report that the filtered medium improved both growth and germination more than algae biomass itself. They propose hormonal signals but did not investigate which compounds were responsible for the observed response. Most recently, Schreiber et al. (2018) grew wheat for 55 days-after-sowing (DAS), longer than previous studies and far past the seedling stage when seed reserve is a source of nutrients to plants. Consistent with previous studies, they found that plants acquired P and N from algae biomass when growing in soil. Growth and elemental uptake varied depending on soil type and algal moisture content. In a nutrient-poor soil type, wheat shoot dry weight was 81 and 90% of mineral fertilized controls with application of wet and dry algae, respectively. In a sand, dried algae biomass was not as effective (87%) compared to wet algae (107%), which even exceeded the mineral fertilizer treatment. Interestingly length and density of the root hairs and root diameters were significantly different between plants treated with algae and mineral fertilizer in the nutrient-poor and sandy soils (Schreiber et al., 2018), suggesting nutrient responses typical of mineral P treatments (Kant et al., 2018) and/or other soil solution signals (Sasse et al., 2018a). Schreiber et al. (2018) suggest that algal biomass nutrients are more slowly acquired than those in mineral fertilizer and nutrient-rich soils. In four contrasting soil types the P fractions fluctuated mostly within the first 3 weeks after addition of either algal biomass or NPK fertilizer, while wheat plants took up qualitatively similar amounts of P from either source (Siebers et al., 2019).

Research to date shows that (1) algae biomass is a fertilizer that will release nutrients over time, and (2) algae biomass may be a source of other compounds that plants respond to in terms of growth. The increasing research into the use of algae as a biofertilizer has not resolved the timing of release and growth responses. Resolution of kinetics of nutrient release from algae biomass and the subsequent uptake by plants is expected to help reveal which forms of P are directly or indirectly accessed by the plant, and thereby improve our prediction about what plant stage and time in cropping seasons responses can be expected by a farmer.

### Question and Approach

Here we ask the question: what is the temporal dynamic of plant growth responses to algal biomass? To address this question, we combine the time resolved wheat leaf growth data from our group (Schreiber et al., 2018) with elemental analyses and other published assumptions to identify critical time points and processes of algal P uptake by wheat. The leaf growth dynamics of wheat plants upon treatment with dried or wet algal biomass or mineral nutrient sources allowed us to propose a model of the timing of P uptake by wheat. We hypothesized that phosphorus from algal biomass is directly available at seedling stage and released gradually over time through to 55 DAS.

## Materials and Methods

Data for this paper were directly from Schreiber et al., 2018; unpublished additional data from Schreiber et al., 2018; transformed from Schreiber et al., 2018; and from other referenced literature sources.

### Plant Biomass and Nutrient Data Analysis From Previous Experiments

#### Data From Schreiber et al., 2018 Experiment

Briefly, Schreiber et al., 2018 quantified wheat (*Triticum aestivum* L., var. Scirocco, KWS SAAT SE, Germany) growth responses to algae biomass (*Chlorella vulgaris* IPPAS C1, grown in a NOVAgreen reactor at the Forschungszentrum Jülich) compared to mineral fertilizer sources in pots, in a glasshouse for 55 days after sowing (DAS) (Treatments in Table 1). The three soils are referred to here as soil 1: nutrient-rich soil (high in organic material); soil 2: nutrient-poor soil (low in available nutrients and high in organic material) and soil 3: sand (low nutrient level and low organic material). Soil 1 + 0 served as positive control, soil 2 + 0 and soil 3 + 0 without fertilizer addition as negative controls for the following nutrient sources. Nutrients were added as Hoagland solution with KH_2_PO_4_ and (NH_4_)H_2_PO_4_ as mineral P [high, + 1x mineral fertilizer (120 mg of P per plant), or low, + 0.1x mineral fertilizer (12 mg P per plant)], or algae [grown without nutrient limitations and harvested at 1 g (DW)^∗^L^–1^]. The algal biomass was either “dried” (spray-dried), or “wet” (with some living cells), and applied as high + 1x dried or wet algae biomass (115 or 130 mg P per plant), or low + 0.1x dried or wet algae biomass (11.5 or 13 mg per plant), for dried and wet algae respectively. All data from the 0.1x nutrient level treatments were not included in the publication of Schreiber et al., 2018, but were part of the experiment. The nomenclature is summarized in Table 1.

**TABLE 1:** List of different treatments.

Name of treatment in text and figures	Soil type	Fertilizer type	P addition [mg/pot]
Soil 1 + 0	nutrient-rich soil	–	0
Soil 2 + 0	nutrient-poor soil	–	0
Soil 2 + 0.1x mineral fertilizer	nutrient-poor soil	mineral fertilizer	12
Soil 2 + 0.1x wet algae biomass	nutrient-poor soil	wet algae biomass	11.5
Soil 2 + 0.1x dried algae biomass	nutrient-poor soil	dried algae biomass	13
Soil 2 + 1x mineral fertilizer	nutrient-poor soil	mineral fertilizer	120
Soil 2 + 1x wet algae biomass	nutrient-poor soil	wet algae biomass	115
Soil 2 + 1x dried algae biomass	nutrient-poor soil	dried algae biomass	130
Soil 3 + 0	sand	–	0
Soil 3 + 0.1x mineral fertilizer	sand	mineral fertilizer	120
Soil 3 + 0.1x wet algae biomass	sand	wet algae biomass	115
Soil 3 + 0.1x + dried algae biomass	sand	dried algae biomass	130

*Summary of different soil types, with soil 1: nutrient-rich soil; soil 2: nutrient-poor soil; or soil 3: sand; and the added types of fertilizers with mineral fertilizer: full strength Hoagland solution; dried algae biomass: spray-dried C. vulgaris IPPAS C1; wet algae biomass: harvested C. vulgaris IPPAS C1 and the contained amount of P in the additions.*

Ten wheat shoots per treatment were repeatedly imaged using an automated crane system (Visser Horti Systems, ‘s-Gravendeel, Netherlands, Nakhforoosh et al., 2016) providing time resolved projected leaf area (PLA) in squared pixels (px^2^) during the entire growth period (greenhouse; 19°C day, 17°C night temperature). At harvest, 55 DAS, leaf area and shoot fresh weight were measured, dry weight of shoots and roots was recorded, and individuals, five per treatment, were analyzed for nutrient content.

Here we transformed and analyzed the Schreiber et al., 2018 data for projected leaf area, dry weight (roots and shoots), P contents measured by ICP-OES (algal biomass, soils, and plant roots and shoots).

#### Data Transformations for Plant Growth and P Uptake Kinetics

Projected leaf area (PLA, in px^2^; Schreiber et al., 2018) was converted to squared centimeters (cm^2^) to compare to other published data (Supplementary Figure S1), using the measured leaf area (at 55 DAS) as described before (Nakhforoosh et al., 2016). We used the resulting linear function to calculate the leaf area for individual plants at each imaging time point. The relative growth rate of the wheat plants was then calculated using the size addition in a certain interval; normalized by the size at the interval beginning as described by Paine et al. (2012). We excluded data for 0.1x nutrient source level, as well as soil 3, to increase comprehensibility and decrease complexity of the figure. Further, we estimated the nutrient uptake per plant per root per time using the linear relationship of nutrient uptake and plant growth under nutrient deprivation (see e.g., De Wit, 1992).

#### Data to Calculate the Origin and Amounts of P

Data from published studies other than Schreiber et al., 2018 were added to estimate sources and forms of P from the soil substrate, the added P source, and the wheat seed. We used that combination to calculate possible contents of the different nutrient sources and sinks of P to compare them at different time points.

Root length at harvest was not measured in Schreiber et al., 2018, and was calculated using published values for specific root length (SRL) of either P limited (Løes and Gahoonia, 2004) or non-limited plants (Figueroa-Bustos et al., 2018) and their respective root dry weight. Potential P limitation of each plant was determined based on the concentration within the tissue (Rashid et al., 2005). In our dataset, the threshold for P limitation was set to <0.17–0.21% P in dry weight per plant.

Basal uptake was defined as the portion of P uptake originating from soil 2 + 0. Per treatment, it was determined using the calculated root length per plant and the P taken up from the plants grown in soil 2 + 0 (Basal uptake_*soil2*_ + 0 = 0.025 mg/m total root length). The root system size before the destructive harvest was estimated by correlating root dry weight and projected leave area (Supplementary Figure S2) at the different time points.

Total P uptake was calculated based on the correlation of final P content and projected leaf area (Supplementary Figure S3). Subtracting the calculated basal uptake from total P uptake per plant at a given time point yielded the P uptake caused by each nutrient source added to soil 2.

An amount of 0.148 mg P was subtracted from each individual plant to account for average seed P (Veneklaas et al., 2012).

### Statistical Analysis of Growth Data

Data were analyzed using Excel (version 2013, Microsoft) and R (Rstudio, version 4.0.2). The values for individual plants were either used directly or the arithmetic mean, and the corresponding error of the mean were calculated to represent the whole sample set. The growth function fitting to the data points was performed in Excel, using *R*^2^ to identify the best fit. *R*^2^ is the squared least square coefficient that will estimate what portion of the variance in the data is explained by the nutrient source factor in linear regressions. Some datasets yielded *R*^2^ values <0.8 and are therefore a rather poor fit, but the best one to represent the data. The individual growth curves of plants were used, both exponential and linear phase, to analyze the differences in growth trajectories between different nutrient sources. Differences between growth trajectories were analyzed using a one-factorized ANOVA and HSD Tukey test for multiple comparison of the means (Lee and Lee, 2018) in R to compare the exponent in the exponential phase and the factor in the linear phase as a measure for the different growth trajectories. Differences between individual time-points were analyzed using the same approach for individual time-points. The growth trajectories and time-points were complemented by the calculation-based relative growth rate - calculated using the leaf area at any given time point, the increase over a defined time span, depending on the measurement points and the occurred increase (Paine et al., 2012). They were compared using a one-factorized ANOVA and HSD Tukey test to compare the RGR between different treatments.

## Results

### Plant Biomass and P Uptake

#### Nutrient Source Influenced Shoot P Content Relative to Biomass

**FIGURE 1:**
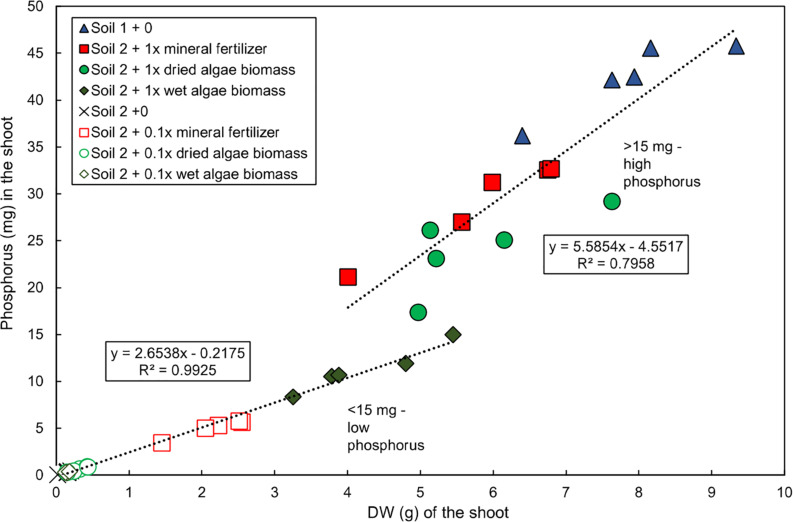
Linear relationship of total P and shoot dry weight of wheat grown with various nutrient sources. Data for total shoot P and shoot dry weight replotted directly from Schreiber et al. (2018). Plants were grown in controlled conditions in the following substrates: soil 1 (nutrient-rich; triangles); soil 2 (nutrient-poor; crosses); or soil 2 with addition of: mineral fertilizer (squares), dried algae biomass (circles) or wet algae biomass (diamonds) either with high (filled forms, 1x) or low amounts (empty forms, 0.1x). Linear correlations were manually fitted to two groups: low P content plants and high P content plants. Each point represents a single plant. *n* = 20 plants for low P group; *n* = 15 for high P group.

Two distinct groups of plants were observed at harvest (55 DAS) based on the relationship between shoot P content and dry weight in the experiment published in Schreiber et al., 2018 (Figure 1). The first group, “low P content,” was grown in soil 2 + 0, soil 2 + 0.1x mineral fertilizer, soil 2 + 0.1x dried algae biomass, soil 2 + 0.1x wet algae biomass, and soil 2 + 1x dried algae biomass. This group had a strong, linear correlation between shoot P content and dry weight (*R*^2^ = 0.9925), averaging 2.65 mg P per g shoot dry weight, and accumulated up to 15 mg of shoot P. The second group, “high P content,” was grown in soil 2 + 1x mineral fertilizer, soil 2 + 1x dried algae biomass, or in soil 1 + 0 (positive control for high nutrient content), had a linear, but less pronounced correlation to shoot dry weight (*R*^2^ = 0.7958), averaging 5.56 mg P per g shoot dry weight, and accumulated up to >45 mg of shoot P.

#### Shoot Growth Rates Depended on Plant Age, Soil Type, and Nutrient Source

**FIGURE 2:**
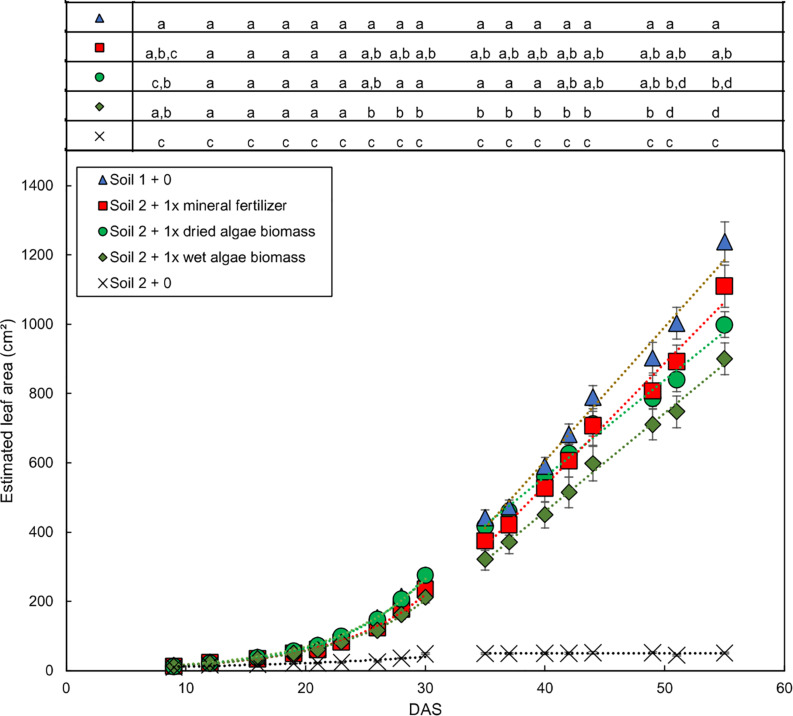
Dynamic development of wheat leaf area dependent on nutrient source mineral fertilizer or algal biomass. Estimated leaf area was calculated by conversion using the relationship shown in Supplementary Figure S1. Leaf area growth over time exhibited an exponential and a linear phase, separated by a line. Plants were grown in controlled conditions in the following substrates: soil 1 (triangles); soil 2 (crosses); or soil 2 with addition of: mineral fertilizer (squares), dried algae biomass (circles) or wet algae biomass (green diamonds) in high (filled forms) amounts. Depicted are means per nutrient sources (*n* = 10 per time point (mineral fertilizer with *n* = 8), error bars represent SE. Statistical differences were analyzed using ANOVA and Tukey multiple comparison of means test *post hoc* test both compared between days (top of the figure) and between growth slopes based on individual growth curves (see Table 2). Different letters indicate significant differences with *p* < 0.05. Equations are fitted to growth curves per phase and their *p*-values (Table 2).

The growth dynamics of wheat shoots over the whole experimental period (Figure 2 for soil 1 and soil 2, Supplementary Figure S4 for soil 3) showed that plants from soil 1 + 0 had the fastest increase in leaf area over time followed by plants grown in soil 2 + 1x dried algae biomass, soil 2 + 1x mineral fertilizer, and soil 2 + 1x wet algae biomass. Plants from soil 2 + 0 had a slower increase in leaf area over time and reached the maximum area, 49 cm^2^, at 30 DAS (linear *R*^2^ = 0.84) compared with 1238 cm^2^ in soil 1 at 55 DAS.

Individual plants followed growth trajectories with two phases (Figure 2). The first phase was exponential (*R*^2^ = 0.99 for all nutrient sources) and lasted for 30 DAS. During phase 1, the growth trajectories were similar and differences between the nutrient sources were marginal. At 26 DAS growth trajectories started to diverge. The second phase was linear (*R*^2^ = 0.99–0.97) and occurred between 30 and 55 DAS. During phase 2, the growth rates (slopes) varied. For example, plants in soil 2 + 1x dried algae biomass had more leaf area at the beginning of phase 2 compared to those with soil 2 + 1x mineral fertilizer, but mineral-fertilized plants gained leaf area much quicker, reaching that of plants grown in soil 1 + 0. Plants with soil 2 + 1x wet algae biomass had the lowest leaf area in phase 2 but grew more quickly than plants grown in soil 2 + 1x dried algae biomass. Statistically significant differences were found between fitted growth functions of the negative control (soil 2 + 0) and all other nutrient sources in both growth phases (adj. *p* < 0.001), between soil 2 + 1x wet algae biomass and soil 2 + 1x dried algae biomass (adj. *p* ≤ 0.001) in the exponential growth phase. Soil 2 + 1x dried algae biomass and soil 2 + 1x mineral fertilizer (adj. *p* = 0.019), as well as soil 2 + 1x dried algae biomass and soil 1 + 0 (adj. *p* ≤ 0.001) differed in the linear phase, there were also differences between soil 2 + 1x wet algae biomass to soil 2 + 1x mineral fertilizer (adj. *p* = 0.044) and soil 2 + 1x wet algae biomass and soil 1 + 0 (adj. *p* ≤ 0.001), when comparing their growth trajectories (Table 2). In summary, algal nutrient sources supported immediate and substantial leaf area growth comparable to the conventional, mineral nutrient source, and this was consistent for soil 2 + 1x dried algae biomass, less consistent for soil 2 + 1x wet algae biomass, and depended on soil type (Figure 2 and Supplementary Figure S4). For example, soil 3 + 1x wet algae biomass led to a higher increase in leaf area compared to other nutrient sources toward the end of the experimental time frame (Supplementary Figure S4), in contrast to treatments in soil 2 (Figure 2).

**TABLE 2:**
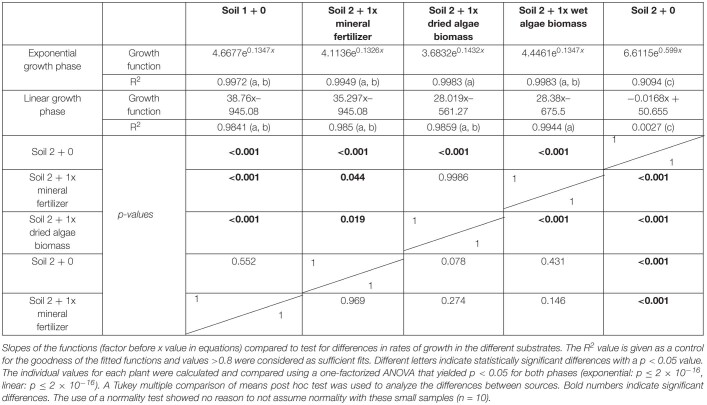
Leaf area of plants grown with mineral fertilizer or different algae forms: Equations for the exponential and linear phases of Figure 3.

*Slopes of the functions (factor before x value in equations) compared to test for differences in rates of growth in the different substrates. The R^2^ value is given as a control for the goodness of the fitted functions and values >0.8 were considered as sufficient fits. Different letters indicate statistically significant differences with a p < 0.05 value. The individual values for each plant were calculated and compared using a one-factorized ANOVA that yielded p < 0.05 for both phases (exponential: p ≤ 2 × 10^–16^, linear: p ≤ 2 × 10^–16^). A Tukey multiple comparison of means post hoc test was used to analyze the differences between sources. Bold numbers indicate significant differences. The use of a normality test showed no reason to not assume normality with these small samples (n = 10).*

**FIGURE 3:**
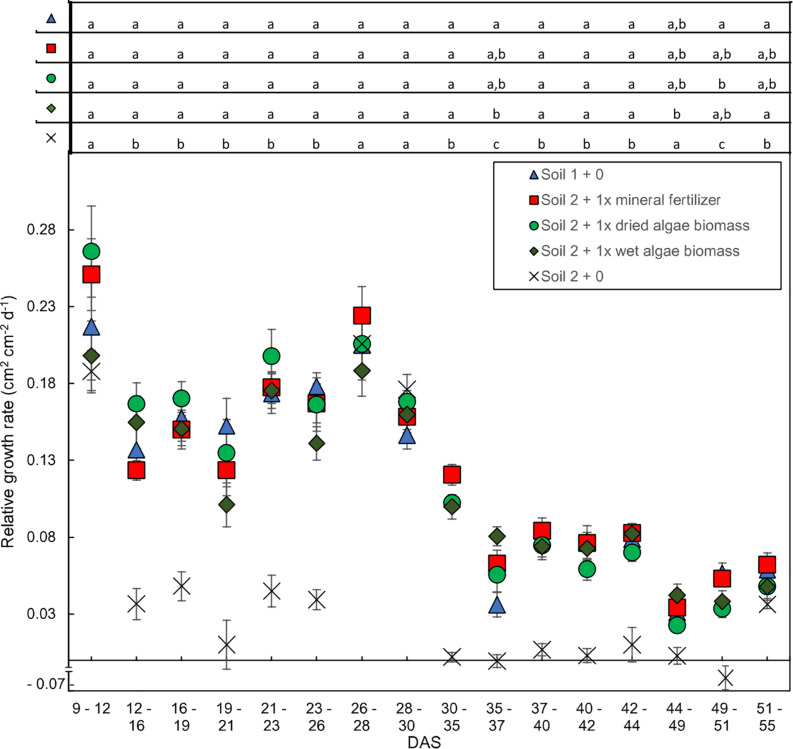
Relative shoot growth rates over time dependent on nutrient source. Relative growth rates are based on change in leaf area per unit leaf area during 2–5-day intervals. Plants were grown in controlled conditions in the following substrates: soil 1 + 0 (triangles); soil 2 + 0 (crosses); or soil 2 with addition of: 1x mineral fertilizer (squares), 1x dried algae biomass (circles) or 1x wet algae biomass (diamonds). Each point represents the mean of *n* = 10 plants with standard error bars. Statistical differences were analyzed using ANOVA and Tukey multiple comparison of means test *post hoc* test both compared between days (top of the figure). Different letters indicate significant differences with *p*** <** 0.05. The break in *y*-axis was inserted manually together with the negative growth value for soil 2 + 0 on interval 49–51 (–0.068 cm^2^ cm^−2^ d^−1^).

The relative leaf area growth rate was calculated to further characterize the time-resolved image data from Schreiber et al., 2018 (Figure 3 presents key treatments). In line with the absolute leaf area (Figure 2), two phases were apparent in the relative leaf area growth rate (Figure 3); an exponential followed by a linear phase, although less pronounced for absolute leaf area. During the exponential phase, before 30 DAS, the plants grew at 0.08–0.28 cm^2^ cm^–2^ day^–1^. All nutrient sources led to similar daily relative growth rates with similar fluctuations over the whole growth period. There was a clear break in the relative growth rate between 28–30 and 35–37 DAS for all nutrient sources marking the transition to the second, linear growth phase. The daily increase of leaf area dropped to less than 0.13 cm^2^ cm^–2^ day^–1^ after 35 DAS in the linear growth phase. Statistical analysis indicated significant differences for all time points comparing soil 2 + 0 to the other treatments, except for days 44–49 were soil 2 + 0 was only different to soil 2 + 1x wet algae biomass fertilized wheat plants. There were also statistical significant differences between soil 1 + 0 and plants grown in soil 2 + 1x wet algae biomass on days 35–37, as well as soil 1 + 0 and soil 2 + 1x dried algae biomass on days 49–51. The last data points (55 DAS) exhibited an increase in growth rate for all nutrient sources again. The exact time of the turning point between growth rates differed between nutrient sources. The plants grown in soil 1 + 0 showed a lower slope than the plants grown in soil 2 + 1x mineral fertilizer, soil 2 + 1x dried algae biomass and soil 2 + 1x wet algae biomass.

### Changes in P Contents of Nutrient Sources and Plants

#### Dynamics of Mineral Fertilizer and Dried and Wet Algae Biomass Had Subtle Differences

**FIGURE 4:**
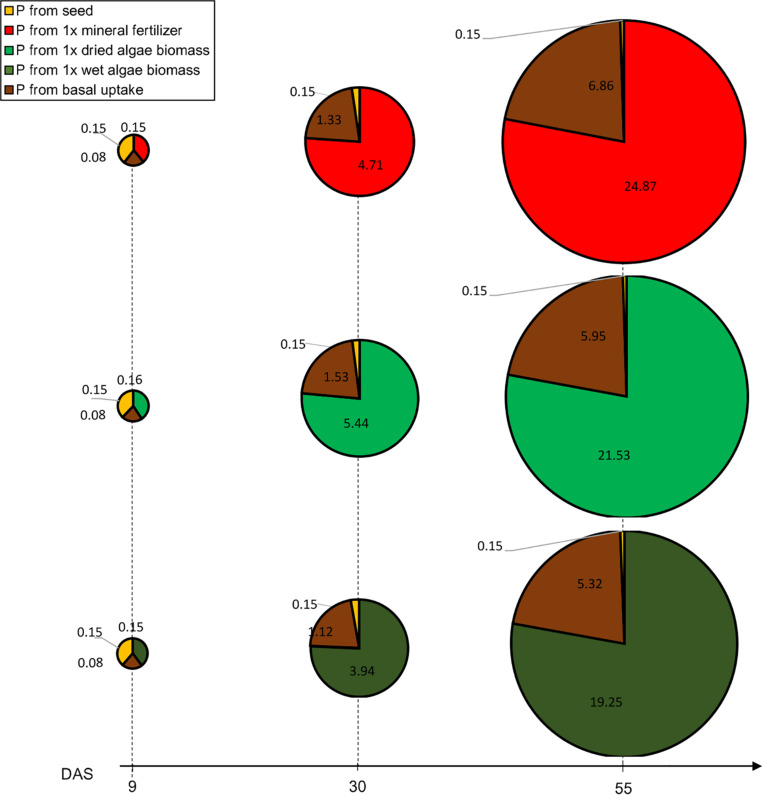
Contribution to P uptake from seed, soil or nutrient source in plants provided with mineral fertilizer or dried algae over time. Values indicate P (in mg) at different time points. P uptake is represented for plants grown in soil 2 + 1x mineral fertilizer (top row), plants grown in soil 2 + 1x dried algae biomass (middle row), and soil 2 + 1x wet algae biomass (bottom row). The area of the circle represents the total P measured at harvest (55 DAS), each circle piece the indicated source. The basal P uptake from the was approximated based on plants grown in soil 2 without addition of nutrients (see section “Materials and Methods”). During the growth period, total P uptake was calculated based on the projected leave area. Values are means per nutrient source (*n* = 5) per time point.

We calculated P uptake from different origins [(seed, soil, nutrient source), Figure 4]. For seed P literature mean values were used; the basal uptake from the soil was based on root dry matter obtained in soil 2 + 0; and the difference to total P was assumed to have originated from the added nutrient source (see section “Materials and Methods”). The basal P uptake was approximated based on a calculated mean root length of 274.6 m (SE = 18.9 m), 238 m (SE = 7.6 m) and 212.9 m (SE = 14.8 m) assessed for all three nutrient sources, soil 2 + 1x mineral fertilizer, soil 2 + 1x dried algae biomass and soil 2 + 1x wet algae biomass, respectively. The mean root dry weight was similar between the three nutrient sources (1.25 g/plant (SE = 0.32 g) for soil 2 + 1x mineral fertilizer, 1.59 g/plant (SE = 0.15 g) for soil 2 + 1x dried algae biomass, 1.34 g/plant (SE = 0.21 g) for soil 2 + 1x wet algae biomass), and therefore their basal uptake was similar as well.

Phosphorus uptake and P contents over time varied among nutrient sources, at three obvious timepoints: beginning of the linear phase at 9 DAS, at the end of the linear phase at 30 DAS, and at the end of the exponential phase at 55 DAS (Figure 4). Initially, the contribution of P uptake from the tested nutrient origins (seed, soil 2, nutrient sources: 1x mineral fertilizer, 1x dried algae biomass, 1x wet algae biomass) between the different nutrient sources was almost identical but changed over time. For all treatments, at 9 DAS the seed, soil 2, and nutrient sources (1x mineral fertilizer, 1x dried algae biomass and 1x wet algae biomass) contributed 40, 20, and 40% to P uptake, respectively, emphasizing that 1x wet and 1x dried algae biomass were a source to plant P from the time of planting in the same amount as mineral fertilizer. At 30 DAS, the contributions changed dramatically with 21% of plant P from basal uptake and 76% from the nutrient sources [(1x mineral fertilizer, 1x dried algae biomass and 1x wet algae biomass), Figure 4]. Finally, at 55 DAS 78% plant P was derived from soil 2 + 1x mineral fertilizer or soil 2 + 1x dried algal biomass. While the P uptake origin was strikingly similar and almost identical, the total P uptake was not. Plants grown on soil 2 + 1x mineral fertilizer took up >4 mg or 13% additional P (total calculated P in mg P/plant: 31.87, SE = 2.19) compared to soil 2 + 1x dried algae biomass (27.63 mg P/plant, SE = 0.88) and >7 mg or 22% of additional P compared to soil 2 + 1x wet algae biomass plants (24.72 mg P/plant, SE = 1.72). Yet, the biomass after 55 DAS was identical for mineral and dry algae-fertilized plants (5.82 g/plant, SE 0.5) and only slightly higher than soil 2 + 1x wet algae biomass (4.23 g/plant, SE = 0.39).

## Discussion

We aimed to answer the question: what is the temporal dynamic of plant growth responses to algal biomass as a sole P source? We conclude that for wheat, in a study that had temporally resolved phenotypic data, phosphorus from algal biomass was directly available and released gradually over time, leading to comparable growth to mineral fertilizer, albeit lower total P uptake.

We used differences in leaf growth dynamics of wheat plants between dried or wet algal or mineral nutrient sources to map out the availability of P. The growth data indicated that, after the seed storage ran out, an initial amount of P was directly available in the early growth phase from all different nutrient sources and could support normal plant growth up until 55 DAS. Algae fertilizer, therefore, was unexpectedly found to be sufficient to support wheat development from the beginning of vegetative growth to beginning of heading, even in marginal soils such as the nutrient-poor soils 2 and 3 used here. We found, however, differences in growth dynamics between fertilized plants and those grown in soil 2 alone, and between dried and wet algae related to uptake dynamics between 30 and 55 DAS. These dynamics are discussed here.

### P Availability Influences Leaf Growth Dynamics

The positive relationship between P in the plant and its biomass that is reported here (Figure 1) is known for wheat and other plant species. For example (Rose et al., 2007) similarly, show for pot experiments with spring wheat that P content and biomass increase up until 60 DAS, and that after a phase of exponential increase, the highest relative P content and dry matter is reached at pre-anthesis.

Similarly, the high and low tissue P contents in Figure 1 match previous data on plant growth under P limitation, and have been attributed to the metabolic costs associated with limited P by e.g., exchanging lipids in membranes (reviewed by Nakamura, 2013). However, the high and low P contents, as related to algal moisture content were unexpected. Dried algae biomass led to approximately twice the total P per plant (mean: 24.2 mg shoot P) compared to wet algae (mean: 11.3 mg shoot P) at 55 DAS. We suggest that the dried algae were putatively dead while the wet contained some living cells, and these nutrient sources led to different shoot growth, and greater dead algal material led to more forms of P available to the plant in the soil. The wet algae biomass living cells may have divided and competed for nutrients with the plant roots, which would change the availability of nutrients in general, as shown for other microorganisms (Kuzyakov and Xu, 2013).

The linearity of shoot P and shoot dry weight that we observed in the plants grown with low nutrient levels and wet algae, was not as strong for the plants grown with high nutritional levels (Figure 1). This may have been attributed to their larger size (greater nutrient demand and/or phenology), or limitation of other nutrients such as nitrogen.

Our analyses of leaf area increase (Figure 2) and relative growth rates (Figure 3) over time followed two-tiered trajectories with an initial exponential phase, followed by a linear phase, similar to that described by Rose et al., 2007. The only exception were plants grown in soil 2 + 0, but they were severely starved for P and also had accelerated maturation (BBCH 51–59 at DAS 55). In the early, exponential growth phase no significant differences between the nutrient sources were found, meaning that similar amounts of nutrients were available and taken up during this phase by plants provided with different nutrient sources. The soil 2 + 1x dried algae biomass and soil 2 + 1x wet algae-fertilized plants did access some nutrients from the soil, as did the plants grown in soil 2 + 0, but these were very limited as reflected in their limited leaf area (Figure 2, see Figure 4 for soil 2 + 1x dried algae biomass or soil 2 + 1x wet algae biomass or soil 2 + 1x mineral fertilizer effect on plants). Literature states that after 2 weeks most of the nutrients from the wheat seed have been consumed (White and Veneklaas, 2012), but growth here after 14 DAS did not differ much between the nutrient sources (Figures 2, 3). After 30 DAS, the relative growth rate changed for all plants, indicating that the plants changed their overall growth mode or maybe even entered a different developmental phase, which should be assessed in further experiments. The *P* values used for our calculations were literature-based and will need experimental verification in the future, because different varieties and growth conditions of mother plants influence the amount and quality of the seed storage in wheat (Kisko et al., 2018).

We assume that root growth was the most relevant growth-limiting parameter for nutrient uptake during the exponential phase, while nutrient release from the added nutrient sources became more dominating once the pot volume had been explored. Root length density (RLD), however, was maximally 0.11 m/cm^3^ for all three nutrient sources. The phosphate uptake rate will, as published by Newman and Andrews, 1973, not yet be limited at RLDs of 0.4 m/cm^3^ by direct root competition. In this context it is worth mentioning that Schreiber et al., 2018 identified differences in root morphology, especially in root hair length, which slightly increased when algae, both wet and dried, were the nutrient sources compared to the mineral fertilizer, allowing the former to access a greater volume of soil given the same RLD. Algal biomass will mainly release phosphate close to the root, because microbial and enzymatic activity will be higher there than in the bulk soil, even though phosphatase activity derived from wheat roots has been shown to be comparably wide spread (Ma et al., 2018), but will also depend on the soil type and moisture content. There could be additional enzyme activity from the biomass itself, because algal cells were shown to express phosphatases themselves (Solovchenko et al., 2019). The RLD will, however, not restrict the uptake of more mobile nutrients, which will probably be accessed independent of the RLD at 30 DAS (Newman and Andrews, 1973). The increment in root biomass (Supplementary Figure S2) between soil 2 + 1x mineral fertilizers and the algal treatments (soil 2 + 1x dried algae biomass or soil 2 + 1x wet algae biomass) is not reflected in an increased leaf area. One possible explanation for this might be a bigger need for soil exploitation in the soil 2 + 1x mineral fertilizer plants due to the differences in P availability. This will be elaborated in detail in the next paragraphs.

### Availability of Algal P Is Temporally and Spatially Different From Mineral P

Our results implicate that, in addition to seed P, an initial amount of P was available early on from all nutrient sources. We assume that residual algal growth media that might still have been attached after a rinse with tap water (Schreiber et al., 2018) was not responsible for the initial growth boost since algae biomass, both dried and wet, led to different growth trends by 30 DAS (Figure 2). Even the effects of additional metabolically active compounds can be ruled out due to this measure (Kholssi et al., 2018). There is some evidence that plants might also be able to take up larger organic molecules and digest them within the roots (Paungfoo-Lonhienne et al., 2010), but the main pathway for P uptake is still considered to be in the form of phosphate using transporters within the outer root membranes; especially root hairs (Gahoonia and Nielsen, 1998). While plants use phytate to store excess P in their cells, algae use polyphosphate to store excess phosphate in granules within storage vacuoles or the cytosol (Aoki and Miyachi, 1964; Ismagulova et al., 2018). Polyphosphate can be the largest portion of P within the algal cells, if they took up excessive amounts of it (Miyachi and Tamiya, 1961b; Solovchenko et al., 2019). Under sufficient growth conditions it might be: 27% phosphate, 57.6% organic compounds and 15.3% polyphosphate (Feng et al., 2016). It has been shown that wheat plants can survive on different organic P sources, e.g., ATP, RNA, and Glucose-1-phosphate, under sterile conditions, but this is limited (Richardson et al., 2000). They can release enzymes and organic acids that can facilitate phosphate degradation of organic compounds and the desorption of phosphate from inorganic compounds or organic matter (Lyu et al., 2016; Yang et al., 2019). Exudation of different components into the rhizosphere will increase the amount available within the rhizosphere by: release of adsorbed phosphate, such as by competition for adsorption sides by the release of organic acids (reviewed in Wang and Lambers, 2019); introduction of enzymes that will speed up the break-down of P containing compounds; or by supplying photosynthates to the rhizosphere microbial population including the mycorrhizal fungi, as carbon is limited in soils (Wang et al., 2016; Sasse et al., 2018b).

**FIGURE 5:**
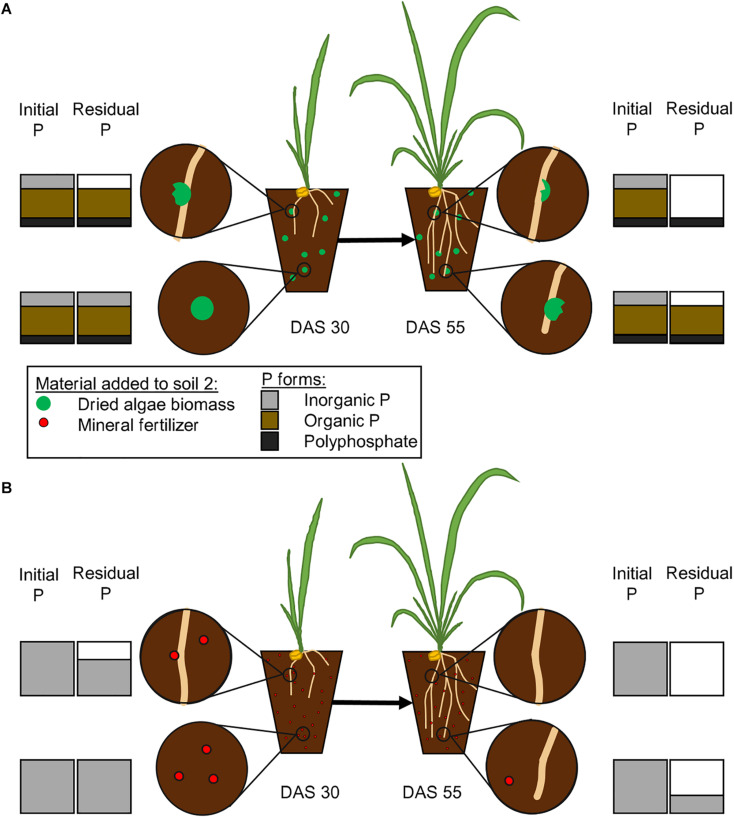
Proposed pattern of phosphate release from mineral fertilizer and dried algae biomass. The initial P bars represent the amount initially added in Schreiber et al., 2018 with either dried algae biomass **(A)** or mineral fertilizer **(B)**. The figure states our hypothesis on how algal biomass will release phosphate from different bound forms **(A)**, including inorganic P, organically bound P and polyphosphates. The algae biomass will release its nutrients close to the roots or in abundance of microbial activity. The plants will take up inorganic portions first (upper left, DAS 30), then facilitate the release of phosphates from organic forms (upper right, DAS 55) and then the polyphosphates. The uptake from mineral fertilizer **(B)** will be less complicated, inorganic phosphate will be taken up, as soon as it is close to the root (upper left, DAS 30) and therefore completely taken up faster (upper right, DAS 55). The amounts of different P forms are estimated from literature Feng et al., 2016.

We developed a time and space resolved concept of P uptake (Figure 5) based on the information that is available on P forms in *Chlorella* sp. (Miyachi and Tamiya, 1961a; Feng et al., 2016) and the ability of wheat plants to take it up. We hypothetically displayed the temporal and spatial differences that will occur between a nutrient source with multiple P forms, such as algae biomass, and a mineral source. Our model on the subsequent release of P from algae biomass and its uptake by plants is based on the assumption that plants will usually take up the cheapest P form, phosphate, first (Turner, 2008), because the more laborious the uptake is, the more energy consuming it becomes. We therefore take into account the enzymatic steps that will be needed to release phosphate to be accessed by the plant (Figure 5), as described before (Turner, 2008; Steidinger et al., 2015).

The mineral fertilizer, especially Hoagland solution, will contain free phosphates and it will, upon application, be available at once. Free phosphates on the other hand can easily become adsorbed, immobilized or leached out of reach, in a time dependent manner, and the longer it is in solution in high amounts the larger the portion that is unavailable for the plant (Shen et al., 2011; Deraoui et al., 2015; Demiraj et al., 2017). Living algae contain a metabolic active P supply, in form of phosphate contained in the cytosol (Solovchenko et al., 2016), while other phosphate groups are bound organically, in order of decreasing amounts: in RNA, phospholipids, DNA, as well as proteins or are stored in inorganic form as polyphosphates (Miyachi and Miyachi, 1961). *Chlorella vulgaris* cell walls are especially robust, and the fast availability of their nutrients is therefore rather surprising (Němcová and Kalina, 2000; Schreiber et al., 2018). The free, inorganic phosphate is, once the algal cell wall is ruptured, assumed to be directly accessible (Figure 5) and similar to the inorganic P from mineral fertilizer also prone to immobilization and leakage. The subsequent release of organic P and polyphosphates into the soil will result in breakdown by either plant or microbial enzymatic activity and the resulting release of phosphate will prolong the P supply of the plant and change the immobilization and adsorption pattern in the soil. The time frame in which free phosphate is abundant will determine the amount of incorporation by microbial activity and adsorption onto soil particles, a subsequent release will therefore reduce the vulnerable time and increase the overall availability. Phospholipids have been shown to have a half-life of almost one day in soils (Zhang et al., 2019), which is similar to what has been described for other compounds. The algal biomass will not have released all its P yet, because as described by Mukherjee et al., 2015, even after 30 days, not all phosphate was released when they incubated algal biomass in soil. They could also show that the involvement of the microbial community is important for the availability of P Mukherjee et al., 2016, which is also an indicator for active facilitation by e.g., enzymes. In addition, polyphosphates can be stable up to 28 days in soil if the pH is between 5.8 and 6.4 (McBeath et al., 2007), unless enzymatic breakdown occurs. If accessed with either phosphatases or phytases, polyphosphate will be degraded within 10 h (Shand and Smith, 1997). The easier accessible compounds such as phosphorylated enzymes and other phosphoproteins, as well as DNA and RNA, might have lost their phosphate groups due to degrading effects prior to polyphosphates. Free phosphates are vulnerable to adsorption onto organic compounds, precipitation, or consumption by microbes, which would transform the available forms into temporarily unavailable ones. The gradual release of phosphate from the larger organic compounds within the algae biomass might prolong these processes and allow a continuous and even increasing supply of nutrients over time. It will be released over time in a specific pattern and our results indicate that algae biomass still supplies available P after 30 DAS up until 55 DAS.

The model presented in Figure 5 and discussed in the paragraph above assumes temporal steps of P breakdown and availability to plants from algae, to explain the quick and continuous growth of wheat compared to mineral, nutrient fertilizer forms. It assumes that plants prefer phosphate to organic forms of P due to the energetics of acquisition. The presented analyses of different P forms and their release as well as their temporally resolved movement from the algal biomass into the plant will be investigated in future experiments.

## Conclusion and Future Experiments

The demonstrated fertilizing potential of algae depends on the release of P compounds readily available to wheat, and some of these forms are taken up from plant establishment. Additional studies of these different compounds and their temporal release will help to understand how we can utilize algae biomass as a fertilizer in the future. An important aspect will be to understand the plant mechanisms behind the P availability from algae for their future application as a fertilizer, along with the role of N in the fertilizing capacity of algal biomass. N volatility and the high requirements of wheat plants makes it another detrimental factor in environmental eutrophication.

## Data Availability Statement

The original contributions presented in the study are included in the article/Supplementary Material, further inquiries can be directed to the corresponding author.

## Author Contributions

LM conducted all analyses, conceived figures, and wrote the manuscript. JK helped to writing the manuscript and the figures. RW helped to construct Figure 5 model and edited the manuscript. CK provided the additional data. SS and UR helped to writing the manuscript. MW conceived the study and helped to writing the manuscript. All authors contributed to the article and approved the submitted version.

## Conflict of Interest

The authors declare that the research was conducted in the absence of any commercial or financial relationships that could be construed as a potential conflict of interest.

## Supplementary Material

Supplementary Figure 1 Correlation of projected leaf area and destructively measured leaf area at 55 DAS. The destructive measurement of leaf area was conducted at 55 DAS. Each data point represents an individual plant, all different nutrient sources are depicted (high and low nutrient level, soil 1 + 0 and soil 2 + 0, + 1x mineral fertilizer + 1x wet algae, + 1x dried algae biomass), with *n* = 10 from each nutrient source and level.

Supplementary Figure 2 Correlation of root dry weight and shoot dry weight to projected leaf area at 55 DAS grown on different nutrient sources and amounts. Destructive measurement of root dry weight shoot weight was conducted 55 DAS. Circles represent the measured leaf area in dependence of shoot dry weight, and squares of root dry weight of all nutrient sources (high and low nutrient level, soil 1 + 0 and soil 2 + 0, + 1x mineral fertilizer + 1x wet algae, + 1x dried algae biomass) from individual plants (*n* = 10 per nutrient source and level).

Supplementary Figure 3 Correlation of plant dry weight and destructively measured P content at 55 DAS. The measurement of P in plant material was conducted at 55 DAS. Each data point represents an individual plant, all different nutrient sources are depicted (high and low nutrient level, soil 1 + 0 and soil 2 + 0, + 1x mineral fertilizer + 1x wet algae, + 1x dried algae biomass), with *n* = 5 from each nutrient source and level.

Supplementary Figure 4 Dynamic development of wheat leaf area dependent on nutrient source mineral fertilizer or algal biomass grown in soil 3. Estimated leaf area was calculated by conversion using the relationship calculated in Figure 1 (data not shown), the used linear equation is *y* = 0.0035*x* (*R*^2^ = 0.9802). Leaf area growth over time exhibited an exponential and a linear phase, separated by a line. Plants were grown in controlled conditions in the following substrates: soil 3 (crosses); or soil 3 with addition of: mineral fertilizer (squares), dried algae biomass (circles) or wet algae biomass (diamonds) in high amounts. Depicted are means per nutrient sources (*n* = 10 per time point, error bars represent SE). Statistical differences were analyzed using ANOVA and Tukey multiple comparison of means test *post hoc* test both compared between days (top of the figure). Different letters indicate significant differences with *p* < 0.05.

## Abbreviations

DAS: days-after-sowing
DW: dry weight
PLA: projected leaf area
RLD: root length density
SRL: specific root length.

## References

[B1] Alobwede E. Leake J. R. Pandhal J. (2019). Circular economy fertilization: testing micro and macro algal species as soil improvers and nutrient sources for crop production in greenhouse and field conditions. *Geoderma* 334 113–123. 10.1016/j.geoderma.2018.07.049

[B2] Aoki S. Miyachi S. (1964). Chromatographic analyses of acid-soluble polyphosphates in chlorella cells. *Plant Cell Physiol.* 5 241–250. 10.1093/oxfordjournals.pcp.a079038

[B3] Bloem E. Albihn A. Elving J. Hermann L. Lehmann L. Sarvi M. (2017). Contamination of organic nutrient sources with potentially toxic elements, antibiotics and pathogen microorganisms in relation to P fertilizer potential and treatment options for the production of sustainable fertilizers: a review. *Sci. Total Environ.* 60 225–242. 10.1016/j.scitotenv.2017.06.274 28692893

[B4] Conley D. J. Paerl H. W. Howarth R. W. Boesch D. F. Seitzinger S. P. Havens K. E. (2009). Ecology – controlling eutrophication: nitrogen and phosphorus. *Science* 323 1014–1015. 10.1126/science.1167755 19229022

[B5] De Wit C. T. (1992). Resource use efficiency in agriculture. *Agric. Syst.* 40 125–151.

[B6] Demiraj E. Brahushi F. Malltezi J. Sulçe S. (2017). Evaluation of phosphorus leaching in an agricultural soil under different soil amendments. *Albanian J. Agric. Sci.* 16 91–100.

[B7] Deraoui N. B. Mekliche L. H. Mihoub A. (2015). Effect of incubation period of phosphorus fertilizer on some properties of sandy soil with low calcareous content. Southern Algeria. *Asian J. Agric. Res.* 9 123–131. 10.3923/ajar.2015.123.131

[B8] Duboc O. Santner J. Golestani Fard A. Zehetner F. Tacconi J. Wenzel W. W. (2017). Predicting phosphorus availability from chemically diverse conventional and recycling fertilizers. *Sci. Total Environ.* 599–600 1160–1170. 10.1016/j.scitotenv.2017.05.054 28511361

[B9] Falkengren-Grerup U. (1995). Interspecies differences in the preference of ammonium and nitrate in vascular plants. *Oecologia* 102 305–311. 10.1007/BF00329797 28306841

[B10] Feng W. Zhu Y. Wu F. He Z. Zhang C. Giesy J. P. (2016). Forms and lability of phosphorus in algae and aquatic macrophytes characterized by solution 31 P NMR coupled with enzymatic hydrolysis. *Sci. Rep.* 6 2–11. 10.1038/srep37164 27849040 PMC5111050

[B11] Figueroa-Bustos V. Palta J. Chen Y. Siddique K. (2018). Characterization of root and shoot traits in wheat cultivars with putative differences in root system size. *Agronomy* 8:109 10.3390/agronomy8070109

[B12] Gahoonia T. S. Nielsen N. E. (1998). Direct evidence on participation of root hairs in phosphorus (32P) uptake from soil. *Plant Soil* 198 147–152. 10.1023/A:1004346412006

[B13] Gimondo J. A. Currey C. J. Jarboe D. H. Gross M. Graves W. R. (2019). Wastewater-grown algae pellets and paste as fertilizers for containerized crops. *HortScience* 54 528–536. 10.21273/HORTSCI13474-18

[B14] Grzesik M. Romanowska-Duda Z. (2014). Improvements in germination, growth, and metabolic activity of corn seedlings by grain conditioning and root application with Cyanobacteria and microalgae. *Polish J. Environ. Stud.* 23 1147–1153.

[B15] Grzesik M. Romanowska-Duda Z. Kalaji H. M. (2017). Effectiveness of cyanobacteria and green algae in enhancing the photosynthetic performance and growth of willow (Salix viminalis L.) plants under limited synthetic fertilizers application. *Photosynthetica* 55 510–521. 10.1007/s11099-017-0716-1

[B16] Güsewell S. (2004). N:P ratios in terrestrial plants: variation and functional significance. *New Phytol.* 164 243–266. 10.1111/j.1469-8137.2004.01192.x 33873556

[B17] Ismagulova T. Shebanova A. Gorelova O. Baulina O. Solovchenko A. (2018). A new simple method for quantification and locating P and N reserves in microalgal cells based on energy-filtered transmission electron microscopy (EFTEM) elemental maps. *PLoS One* 13:e0208830. 10.1371/journal.pone.0208830 30533056 PMC6289464

[B18] Kant J. Ishizaki T. Pariasca-Tanaka J. Rose T. Wissuwa M. Watt M. (2018). “Phosphorus efficient phenotype of rice,” in *Rice Crop – Current Developments*, eds Iqbal A. Shah F. Khan Z. (London: InTech).

[B19] Kholssi R. Marks E. A. N. Miñón J. Montero O. Debdoubi A. Rad C. (2018). Biofertilizing effect of *Chlorella sorokiniana* suspensions on wheat growth. *J. Plant Growth Regul.* 38 644–649. 10.1007/s00344-018-9879-7

[B20] Kisko M. Shukla V. Kaur M. Bouain N. Chaiwong N. Lacombe B. (2018). Phosphorus transport in arabidopsis and wheat: emerging strategies to improve P pool in seeds. *Agriculture* 8:27 10.3390/agriculture8020027

[B21] Kuzyakov Y. Xu X. (2013). Competition between roots and microorganisms for nitrogen: mechanisms and ecological relevance. *New Phytol.* 198 656–669. 10.1111/nph.12235 23521345

[B22] Lee S. Lee D. K. (2018). What is the proper way to apply the multiple comparison test? *Korean J. Anesthesiol.* 71 353–360. 10.4097/kja.d.18.00242 30157585 PMC6193594

[B23] Løes A. K. Gahoonia T. S. (2004). Genetic variation in specific root length in Scandinavian wheat and barley accessions. *Euphytica* 137 243–249. 10.1023/B:EUPH.0000041587.02009.2e

[B24] Lyu Y. Tang H. Li H. Zhang F. Rengel Z. Whalley W. R. (2016). Major crop species show differential balance between root morphological and physiological responses to variable phosphorus supply. *Front. Plant Sci.* 7:1939. 10.3389/fpls.2016.01939 28066491 PMC5174099

[B25] Ma X. Zarebanadkouki M. Kuzyakov Y. Blagodatskaya E. Pausch J. Razavi B. S. (2018). Spatial patterns of enzyme activities in the rhizosphere: effects of root hairs and root radius. *Soil Biol. Biochem.* 118 69–78. 10.1016/j.soilbio.2017.12.009

[B26] MacDonald G. K. Bennett E. M. Potter P. A. Ramankutty N. (2011). Agronomic phosphorus imbalances across the world’s croplands. *Proc. Natl. Acad. Sci.U.S.A.* 108 3086–3091. 10.1073/pnas.1010808108 21282605 PMC3041096

[B27] McBeath T. M. Lombi E. McLaughlin M. J. Bünemann E. K. (2007). Polyphosphate-fertilizer solution stability with time, temperature, and pH. *J. Plant Nutr. Soil Sci.* 170 387–391. 10.1002/jpln.200625166

[B28] Miyachi S. Kanai R. Mihara S. Miyachi S. Aoki S. (1964). Metabolic roles of inorganic polyphosphates in chlorella cells. *Biochim. Biophys. Acta Gen. Subj.* 93 625–634. 10.1016/0304-4165(64)90345-9 14263160

[B29] Miyachi S. Miyachi S. (1961). Modes of formation of phosphate compounds and their turnover in chlorella cells during the process of life cycle as studied by the technique of synchronous culture. *Plant Cell Physiol.* 2 415–424.

[B30] Miyachi S. Tamiya H. (1961a). Distribution and turnover of phosphate compounds in growing chlorella cells. *Plant Cell Physiol.* 2 405–414. 10.1093/oxfordjournals.pcp.a077695

[B31] Miyachi S. Tamiya H. (1961b). Some observations on the phosphorus metabolism in growing Chlorella cells. *Biochim. Biophys. Acta* 46 200–202. 10.1002/mrm.24251 13771481

[B32] Moudříková Š Nedbal L. Solovchenko A. Mojzeš P. (2017). Raman microscopy shows that nitrogen-rich cellular inclusions in microalgae are microcrystalline guanine. *Algal Res.* 23 216–222. 10.1016/j.algal.2017.02.009

[B33] Mukherjee C. Chowdhury R. Ray K. (2015). Phosphorus recycling from an unexplored source by polyphosphate accumulating microalgae and cyanobacteria-a step to phosphorus security in agriculture. *Front. Microbiol.* 6:1421. 10.3389/fmicb.2015.01421 26733966 PMC4686675

[B34] Mukherjee C. Chowdhury R. Sutradhar T. Begam M. Basak S. K. Ray K. (2016). Parboiled rice effluent: a wastewater niche for microalgae and cyanobacteria with growth coupled to comprehensive remediation and phosphorus biofertilization. *Algal Res.* 19 225–236. 10.1016/j.algal.2016.09.009

[B35] Mulbry W. Westhead E. K. Pizarro C. Sikora L. (2005). Recycling of manure nutrients: use of algal biomass from dairy manure treatment as a slow release fertilizer. *Bioresour. Technol.* 96 451–458. 10.1016/j.biortech.2004.05.026 15491826

[B36] Nakamura Y. (2013). Phosphate starvation and membrane lipid remodeling in seed plants. *Prog. Lipid Res.* 52 43–50. 10.1016/j.plipres.2012.07.002 22954597

[B37] Nakhforoosh A. Bodewein T. Fiorani F. Bodner G. (2016). Identification of water use strategies at early growth stages in durum wheat from shoot phenotyping and physiological measurements. *Front. Plant Sci.* 7:1155. 10.3389/fpls.2016.01155 27547208 PMC4974299

[B38] Němcová Y. Kalina T. (2000). Cell wall development, microfibril and pyrenoid structure in type strains of Chlorella vulgaris, C. kessleri, C. sorokiniana compared with C. luteoviridis (Trebouxiophyceae, Chlorophyta). *Algol. Stud. für Hydrobiol.* 100(Suppl.) 95–105. 10.1127/algol_stud/100/2000/95

[B39] Newman E. I. Andrews R. E. (1973). Uptake of phosphorus and potassium in relation to root growth and root density. *Plant Soil* 38 49–69. 10.1007/BF00011217

[B40] Paine C. E. T. Marthews T. R. Vogt D. R. Purves D. Rees M. Hector A. (2012). How to fit nonlinear plant growth models and calculate growth rates: an update for ecologists. *Methods Ecol. Evol.* 3 245–256. 10.1111/j.2041-210X.2011.00155.x

[B41] Paungfoo-Lonhienne C. Lonhienne T. G. A. Mudge S. R. Schenk P. M. Christie M. Carroll B. J. (2010). DNA is taken up by root hairs and pollen, and stimulates root and pollen tube growth. *Plant Physiol.* 153 799–805. 10.1104/pp.110.154963 20388669 PMC2879792

[B42] Paungfoo-Lonhienne C. Lonhienne T. G. A. Rentsch D. Robinson N. Christie M. Webb R. I. (2008). Plants can use protein as a nitrogen source without assistance from other organisms. *Proc. Natl. Acad. Sci. U.S.A.* 105 4524–4529. 10.1073/pnas.0712078105 18334638 PMC2393761

[B43] Piotrowski K. Romanowska-Duda Z. Grzesik M. (2016). How biojodis and cyanobacteria alleviate the negative in fl uence of predicted environmental constraints on growth and physiological activity of corn plants. *Pol. J. Environ. Stud.* 25 741–751. 10.15244/pjoes/60894

[B44] Powell N. Shilton A. N. Pratt S. Chisti Y. (2008). Factors influencing luxury uptake of phosphorus by microalgae in waste stabilization ponds. *Environ. Sci. Technol.* 42 5958–5962. 10.1021/es703118s 18767651

[B45] Rahman M. M. Liu Y. H. Kwag J. H. Ra C. S. (2011). Recovery of struvite from animal wastewater and its nutrient leaching loss in soil. *J. Hazard Mater.* 186 2026–2030. 10.1016/j.jhazmat.2010.12.103 21236571

[B46] Rashid A. Awan Z. I. Ryan J. (2005). Diagnosing phosphorus deficiency in spring wheat by plant analysis: proposed critical concentration ranges. *Commun. Soil Sci. Plant Anal.* 36 609–622. 10.1081/CSS-200043299

[B47] Richardson A. E. Hadobas P. A. Hayes J. E. (2000). Acid phosphomonoesterase and phytase activities of wheat (*Triticum aestivum* L.) roots and utilization of organic phosphorus substrates by seedlings grown in sterile culture. *Plant Cell Environ.* 23 397–405. 10.1046/j.1365-3040.2000.00557.x

[B48] Rose T. J. Rengel Z. Ma Q. Bowden J. W. (2007). Differential accumulation patterns of phosphorus and potassium by canola cultivars compared to wheat. *J. Plant Nutr. Soil Sci.* 170 404–411. 10.1002/jpln.200625163

[B49] Safi C. Zebib B. Merah O. Pontalier P.-Y. Vaca-Garcia C. (2014). Morphology, composition, production, processing and applications of Chlorella vulgaris: a review. *Renew. Sustain. Energy Rev.* 35 265–278. 10.1016/j.rser.2014.04.007

[B50] Sasse J. Kant J. Cole B. J. Klein A. P. Arsova B. Schlaepfer P. (2018a). Multi-lab EcoFAB study shows highly reproducible physiology and depletion of soil metabolites by a model grass. *bioRxiv* [preprint] 10.1101/435818 PMC6519027 30585637

[B51] Sasse J. Martinoia E. Northen T. (2018b). Feed your friends: do plant exudates shape the root microbiome? *Trends Plant Sci.* 23 25–41. 10.1016/j.tplants.2017.09.003 29050989

[B52] Schreiber C. Schiedung H. Harrison L. Briese C. Ackermann B. Kant J. (2018). Evaluating potential of green alga Chlorella vulgaris to accumulate phosphorus and to fertilize nutrient-poor soil substrates for crop plants. *J. Appl. Phycol.* 30 2827–2836. 10.1007/s10811-018-1390-9

[B53] Shand C. A. Smith S. (1997). Enzymatic release of phosphate from model substrates and P compounds in soil solution from a peaty podzol. *Biol. Fertil. Soils* 24 183–187. 10.1007/s003740050229

[B54] Shen J. Yuan L. Zhang J. Li H. Bai Z. Chen X. (2011). Phosphorus dynamics: from soil to plant. *Plant Physiol.* 156 997–1005. 10.1104/pp.111.175232 21571668 PMC3135930

[B55] Shilton A. N. Powell N. Guieysse B. (2012). Plant based phosphorus recovery from wastewater via algae and macrophytes. *Curr. Opin. Biotechnol.* 23 884–889. 10.1016/j.copbio.2012.07.002 22889679

[B56] Siebers N. Hofmann D. Schiedung H. Landsrath A. Ackermann B. Gao L. (2019). Towards phosphorus recycling for agriculture by algae: soil incubation and rhizotron studies using 33 P-labeled microalgal biomass. *Algal Res.* 43:101634 10.1016/j.algal.2019.101634

[B57] Solovchenko A. Khozin-Goldberg I. Selyakh I. Semenova L. Ismagulova T. Lukyanov A. (2019). Phosphorus starvation and luxury uptake in green microalgae revisited. *Algal Res* 43:101651 10.1016/j.algal.2019.101651

[B58] Solovchenko A. Verschoor A. M. Jablonowski N. D. Nedbal L. (2016). Phosphorus from wastewater to crops: an alternative path involving microalgae. *Biotechnol. Adv.* 34 550–564. 10.1016/j.biotechadv.2016.01.002 26795876

[B59] Steidinger B. S. Turner B. L. Corrales A. Dalling J. W. (2015). Variability in potential to exploit different soil organic phosphorus compounds among tropical montane tree species. *Funct. Ecol.* 29 121–130. 10.1111/1365-2435.12325

[B60] Turnbull M. H. Schmidt S. Erskine P. D. Richards S. Stewart G. R. (1996). Root adaptation and nitrogen source acquisition in natural ecosystems. *Tree Physiol.* 16 941–948. 10.1093/treephys/16.11-12.941 14871787

[B61] Turner B. L. (2008). Resource partitioning for soil phosphorus: a hypothesis. *J. Ecol.* 96 698–702. 10.1111/j.1365-2745.2008.01384.x

[B62] Veneklaas E. J. Lambers H. Bragg J. Finnegan P. M. Lovelock C. E. Plaxton W. C. (2012). Opportunities for improving phosphorus-use efficiency in crop plants. *New Phytol.* 195 306–320. 10.1111/j.1469-8137.2012.04190.x 22691045

[B63] Wang Y. Krogstad T. Clarke J. L. Hallama M. Øgaard A. F. Eich-Greatorex S. (2016). Rhizosphere organic anions play a minor role in improving crop species’ ability to take up residual phosphorus (P) in agricultural soils low in P availability. *Front. Plant Sci.* 7:1664. 10.3389/fpls.2016.01664 27872635 PMC5097927

[B64] Wang Y. Lambers H. (2019). Root-released organic anions in response to low phosphorus availability: recent progress, challenges and future perspectives. *Plant Soil* 447 135–156. 10.1007/s11104-019-03972-8

[B65] White P. J. Veneklaas E. J. (2012). Nature and nurture: the importance of seed phosphorus content. *Plant Soil* 357 1–8. 10.1007/s11104-012-1128-4

[B66] Yang X. Chen X. Yang X. (2019). Effect of organic matter on phosphorus adsorption and desorption in a black soil from Northeast China. *Soil Tillage Res.* 187 85–91. 10.1016/j.still.2018.11.016

[B67] Zhang Y. Zheng N. Wang J. Yao H. Qiu Q. Chapman S. J. (2019). High turnover rate of free phospholipids in soil confirms the classic hypothesis of PLFA methodology. *Soil Biol. Biochem.* 135 323–330. 10.1016/j.soilbio.2019.05.023

