# Supplementary material for: Wheat Can Access Phosphorus From Algal Biomass as Quickly and Continuously as From Mineral Fertilizer
Source: Front Plant Sci. 2021 Jan 28;12:631314. doi: 10.3389/fpls.2021.631314 (PMC7879783; doi:10.3389/fpls.2021.631314)
Supplement: Supplementary Figure 1 — Correlation of projected leaf area and destructively measured leaf area at 55 DAS. The destructive measurement of leaf area was conducted at 55 DAS. Each data point represents an individual plant, all different nutrient sources are depicted (high and low nutrient level, soil 1 + 0 and soil 2 + 0, + 1x mineral fertilizer + 1x wet algae, + 1x dried algae biomass), with n = 10 from each nutrient source and level. [file Data_Sheet_1.PDF]

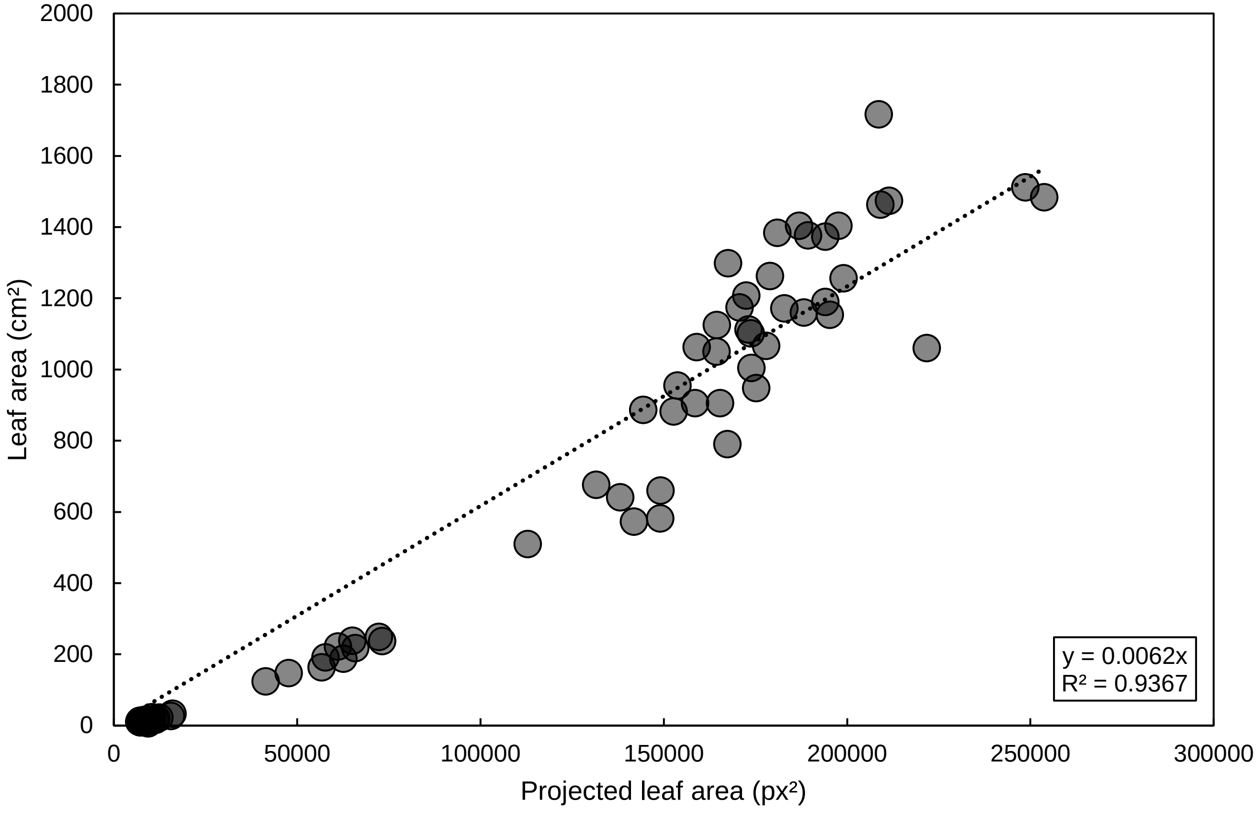

Figure S1: Correlation of projected leaf area and destructively measured leaf area at 55 DAS. The destructive measurement of leaf area was conducted at 55 DAS. Each data point represents an individual plant, all different nutrient sources are depicted (high and low nutrient level, soil 1 and soil 2, + mineral fertilizer + wet algae, + dried algae biomass), with n= 10 from each nutrient source and level.

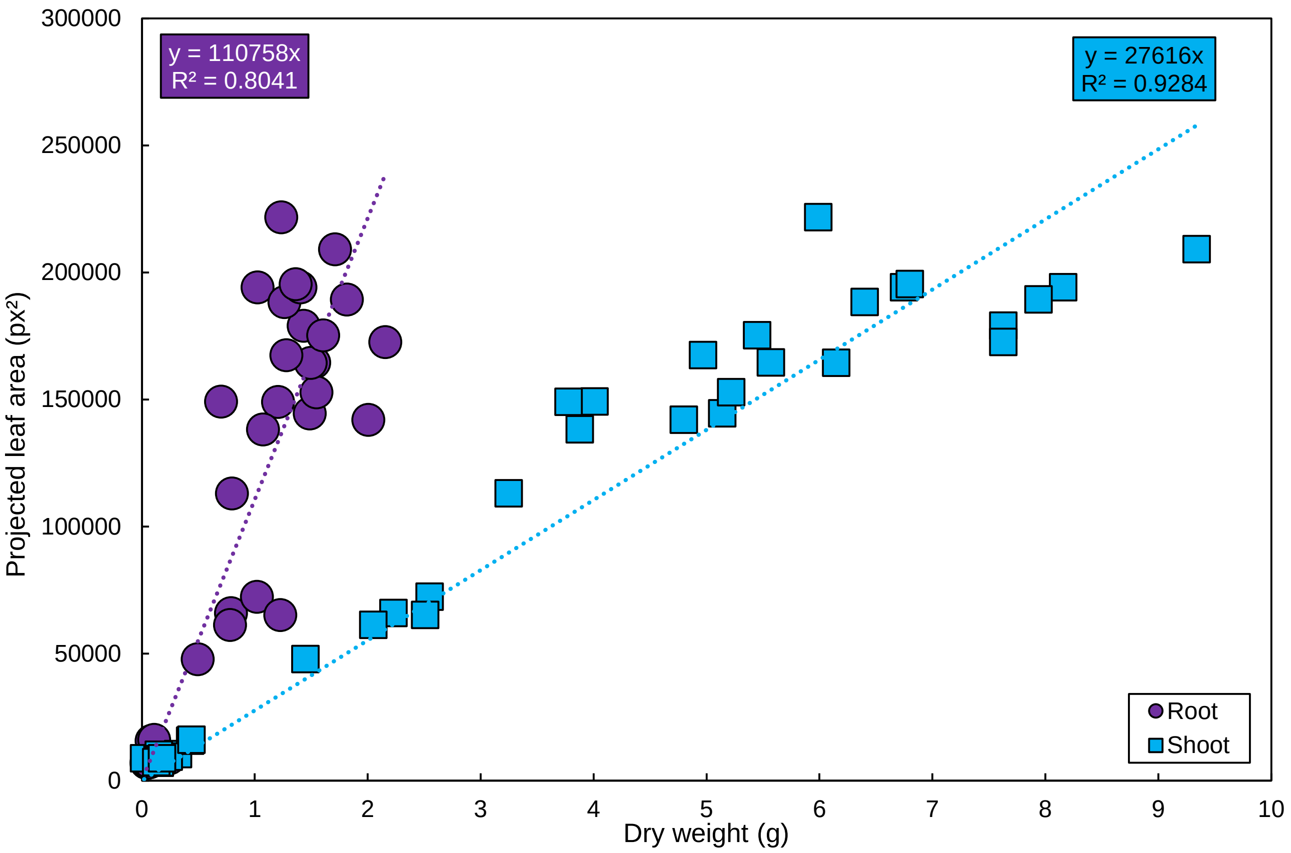

Figure S2: Correlation of root dry weight and shoot dry weight to projected leaf area at 55 DAS grown on different nutrient sources and amounts. Destructive measurement of root dry weight, shoot weight was conducted 55 DAS. Circles represent the measured leaf area in dependence of shoot dry weight, and squares of root dry weight of all nutrient sources (high and low nutrient level, soil 1 and soil 2, + mineral fertilizer + wet algae, + dried algae biomass) from individual plants (n=10 per nutrient source and level).

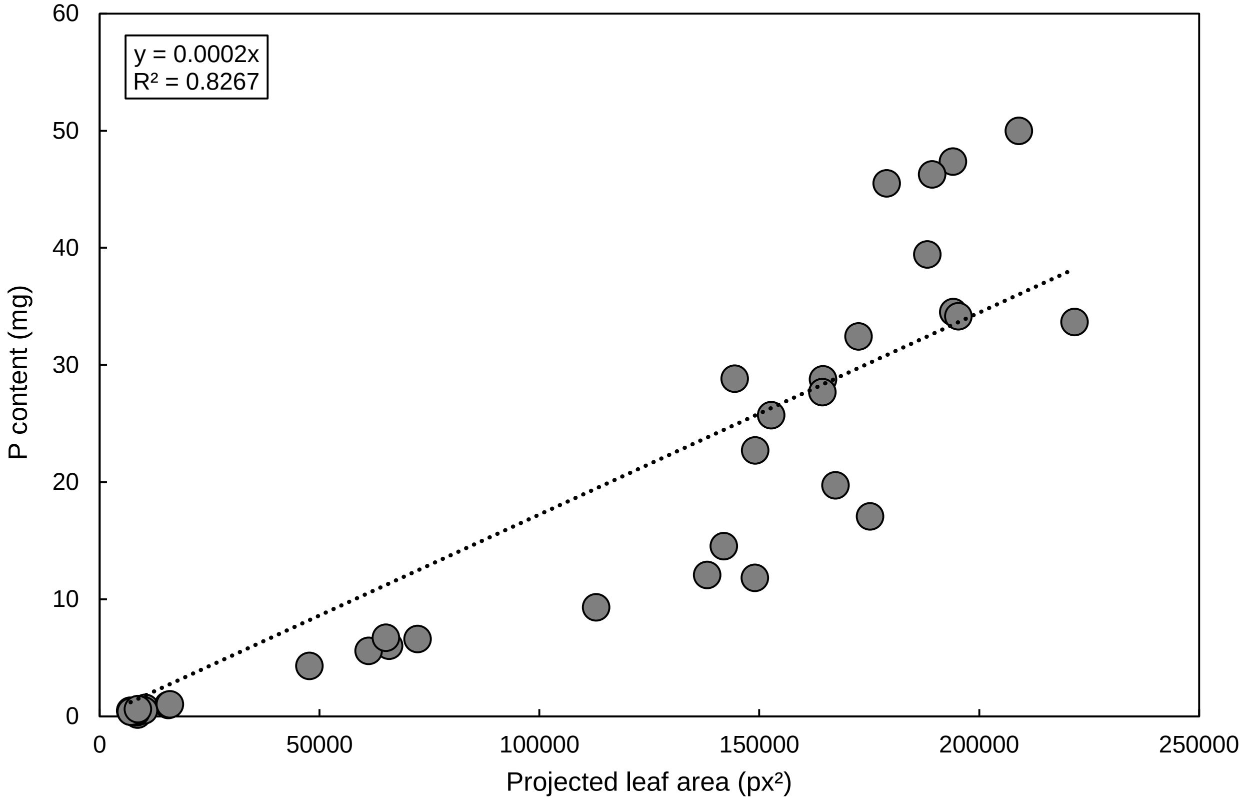

Figure S3: Correlation of PLA and destructively measured P content at 55 DAS. The destructive measurement of P was conducted at 55 DAS. Each data point represents an individual plant, all different nutrient sources are depicted (high and low nutrient level, soil 1 and soil 2, + mineral fertilizer + wet algae, + dried algae biomass), with n= 5 from each nutrient source and level.

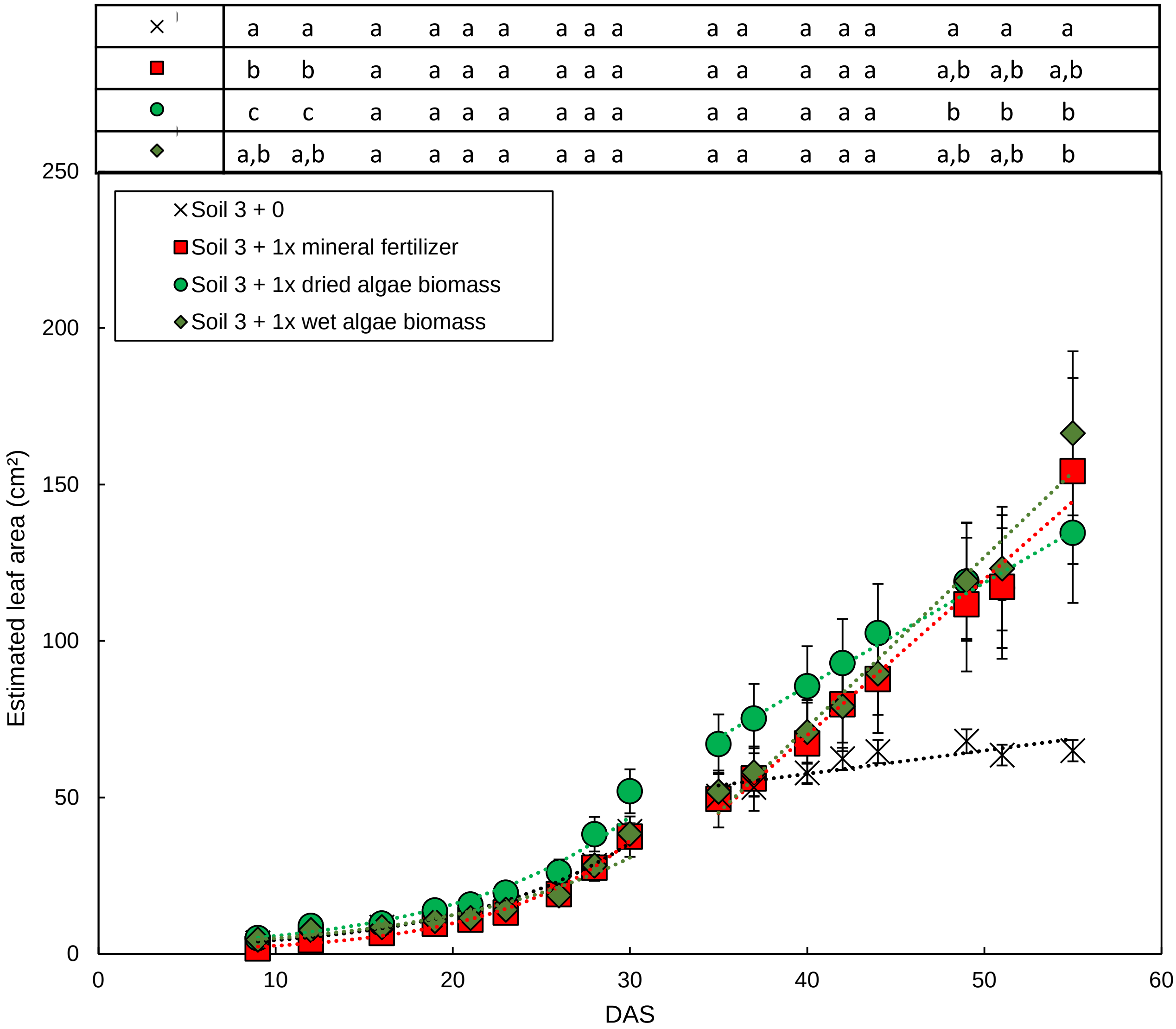

Figure S4: Dynamic development of wheat leaf area dependent on nutrient source mineral fertilizer or algal biomass grown in soil 3. Estimated leaf area was calculated by conversion using the relationship calculated in Figure 1 (data not shown), the used linear equation is $y = 0.0035x$ ($R^2 = 0.9802$). Leaf area growth over time exhibited an exponential and a linear phase, separated by a line. Plants were grown in controlled conditions in the following substrates: soil 3 (crosses); or soil 3 with addition of: mineral fertilizer (squares), dried algae biomass (circles) or wet algae biomass (diamonds) in high amounts. Depicted are means per nutrient sources (n= 10 per time point, error bars represent SE). Statistical differences were analysed using ANOVA and Tukey multiple comparison of means test post-hoc test both compared between days (top of the figure). Different letters indicate significant differences with $p < 0.05$.
